# Case Report: Intensity-modulated radiotherapy combined with immunotherapy for intramedullary spinal cord metastases of lung adenocarcinoma

**DOI:** 10.3389/fonc.2025.1367346

**Published:** 2025-03-10

**Authors:** Yingying He, Fei Xie, Tianli He, Zhou Zhou, Zhaohong Chen, Lixing Jiang, Wei Hu

**Affiliations:** ^1^ Oncology Department, Deyang People’s Hospital, Deyang, China; ^2^ Radiotherapy Oncology Department, Changxing Campus (Changxing People's Hospital), Second Affiliated Hospital of Zhejiang University School of Medicine, Changxing, China

**Keywords:** intramedullary spinal cord metastases, intestinal obstruction, intensity modulated radiotherapy, immunotherapy, pneumonia

## Abstract

Intramedullary spinal cord metastases (ISCMs) are rare in clinical practice and their presentation is usually an unfavorable sign with a median overall survival (mOS) of 3-4 months after diagnosis. Due to their rarity, heterogeneity and rapid progression, clinicians have few satisfactory guidelines or optimal management for ISCMs. Herein, we share a clinical experience of intensity-modulated radiotherapy (IMRT) combined with immunotherapy (IO) for ISCMs from lung adenocarcinoma (LUAD) that achieved a relatively high quality of life for 10 months, which has not been previously reported.

## 1 Introduction

ISCMs from solid tumors are rare events, with an incidence of approximately 0.1%-2% (1). ISCMs from lung cancer (ISCMs-LC) are the predominant type, accounting for 42.4%-67.21% (1, 2), of which, small cell lung cancer (SCLC) accounts for 39.1%, followed by LUAD (25.1%) and squamous cell carcinoma (10.6%) (3). Most patients with ISCMs have concomitant brain metastases (BM), and even leptomeningeal metastases, reflecting that tumor cells can metastasize to the meninges through cerebrospinal fluid (CSF) and then spread to the spinal cord parenchyma or spread directly to the subarachnoid space and spinal cord parenchyma through the nerve roots or CSF (4). The clinical presentation of ISCMs is similar to that spinal epidural metastases, but a distinguishing feature is a Brown–Sequard syndrome (also known as syndrome of hemilateral spinal cord injury syndrome) or asymmetric myelopathy, which is seen in half of patients with ISCMs but only 3% of patients with spinal epidural metastases (5). Characteristic symptoms of ISCMs include paresthesia, sensory loss, and leg weakness with rapid deterioration (2).

The prognosis of ISCMs is extremely poor, as their presence often indicates end-stage cancer, and mOS after diagnosis is approximately 3-4 months (4, 6). Although some clinicians reported experience with surgery (1, 7, 8), radiotherapy (9, 10), chemotherapy (11, 12), and targeted therapy (13), optimal treatment modalities for ISCMs have not been established due to their rarity, heterogeneity, and rapid progression.

Nowadays, IO has been the first-line therapy for advanced LUAD with negative driver gene mutations. As radiotherapy combined with IO could stimulate a robust systemic immune response and improve clinical outcomes (14), radiotherapy combined with IO has been used in clinical settings for locally advanced esophagus cancer (15), nasopharyngeal carcinoma (16) and lung cancer (17). In addition, radiotherapy has shown promising local control with overall acceptable toxicity in some thoracic pathologies with a poor prognosis, such as multiple relapsed malignant pleural mesothelioma (18), extensive-stage small cell lung cancer (ES-SCLC) (19), lung cancer-related BM (20, 21). However, there are still no reports of radiotherapy combined with IO for ISCMs. Here, we report a case of regression of ISCMs treated with IMRT combined with IO.

## 2 Case presentation

A 65-year-old gentleman with a heavy smoking history underwent a thoracic computed tomography (CT) scan in June 2021, which showed an 85 mm mass in the left upper lung with metastases to the right lung, mediastinal and left hilar lymph nodes. He then underwent endobronchial ultrasound (EBUS) and transbronchial needle aspiration (TBNA) biopsy. Hematoxylin-eosin (HE) staining and immunohistochemical (IHC) indicators such as TTF-1 (+), CK-7 (+), Cg-A (±), syn (+), P40 (-) supported the diagnosis of LUAD with neuroendocrine differentiation. Furthermore, EGFR mutation, ALK rearrangement and KRAS mutation were negative. Based on the International Association for the Study of Lung Cancer (IASLC, 8th edition TNM staging system), the diagnosis was LUAD with negative driver gene mutations (T4N2M1a, stage IVa). Atezolizumab (a programmed cell death ligand-1 blocker) 1200 mg in combination with paclitaxel liposome 240 mg was administered every 3 weeks. After 6 cycles of treatment, a CT scan in October 2021 showed multiple BM, and whole brain radiotherapy (WBRT) was administered with a total dose of 30 Gy/10 fractions. Atezolizumab monotherapy was continued every 3 weeks.

**Figure 1:**
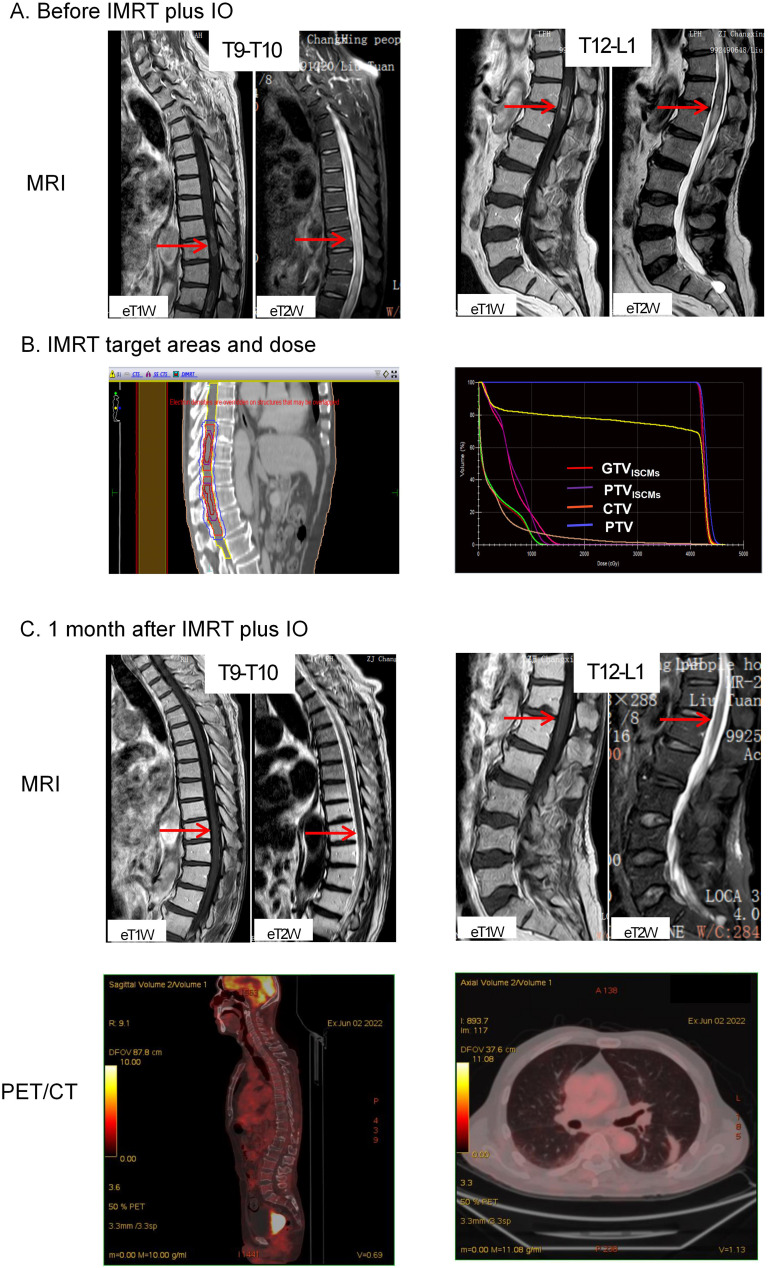
Imaging of tumor lesions before and after treatment. **(A)** MRI before IMRT plus IO showed that the signal of the spinal cord was not homogeneous, with foci of enhancement (indicated by red arrows) in the spinal cord at the T9-T10 and T12-L1 levels. **(B)** IMRT target areas in the median sagittal section (left) and total volume DVH (right). The bright red line is GTV_ISCMs_, the purple line is PTV_ISCMs_, the orange line is CTV and the blue line is PTV. The 100% radiation volume of GTV_ISCMs_, PTV_ISCMs_, CTV and PTV reached the dose of 40Gy, and the 95% radiation volume of PTV reached the dose of 42Gy. **(C)** MRI after IMRT plus IO treatment showed that the enhancement signal of the original lesions at T9-10 and T12-L1 levels decreased as an oedema signal (indicated by red arrows). In addition, PET/CT showed no abnormal FDG uptake throughout the spinal cord, mediastinum and both lungs. IMRT, intensity-modulated radiotherapy; IO, immunotherapy; MRI, magnetic resonance imaging; PET/CT, positron emission tomography/computed tomography; FDG, ^18^F-fluorodeoxyglucose. ISCMs, intramedullary spinal cord metastases; DVH, dose-volume histogram; GTV_ISCMs_, gross tumor volume of ISCMs; PTV_ISCMs_, planning target volume of ISCMs; CTV, clinical target volume; PTV, planning target volume.

In March 2022, he felt bloated and had difficulty passing urine and stool. The symptoms gradually worsened, and in April 2022, he developed intestinal obstruction and urinary retention, which were only treated symptomatically with catheterisation and enema. Less than a week later, he developed bilateral lower limb pain with muscle weakness and was admitted to the Changxing Campus of the Second Affiliated Hospital of Zhejiang University School of Medicine (SAHZU). Physical examination revealed bilateral hypoesthesia below the inguinal ligament, grade 3 muscle strength of the left lower extremity, grade 4 muscle strength of the right lower extremity, and hyporeflexia of the knee. MRI showed that the spinal cord signal was not homogeneous, with foci of enhancement in the spinal cord at the T9-T10 and T12-L1 levels (**Figure 1A**). An online multidisciplinary collaborative discussion was held for him, involving oncology, imaging, neurology, neurosurgery and radiotherapy from Changxing Campus and Deyang People’s Hospital. ISCMs was diagnosed based on the history of LUAD, the rapid progression of neurological symptoms and the appearance on MRI. A comprehensive strategy was then tailored to relieve cancer pain and improve mobility with neurological function. Based on symptomatic treatment with dexamethasone and oxycodone, IMRT combined with atezolizumab was administered to achieve local control and activate systemic antitumor immunity. The detailed antitumor regimen consisted of 1200 mg atezolizumab monotherapy every 3 weeks and concurrent IMRT targeting ISCMs lesions with the 95% planning target volume (PTV) dose reaching 42Gy/21 fractions (**Figure 1B**).

Two weeks after starting IMRT, symptoms improved, including partial recovery of muscle strength in both lower extremities and relief of pain in the lower extremities. By the end of IMRT, he had regained normal urination and bowel movements. In addition, muscle strength in both lower extremities was almost back to normal and pain medication was no longer required. At this point, he had only a little numbness in his lower limbs, which did not affect his ability to walk independently. One month after completion of IMRT, a repeat MRI was performed, which showed that the enhancing lesions in the spinal cord at the T9-T10 and T12-L1 levels had disappeared (**Figure 1C**). In addition, an ^18^F-fluorodeoxyglucose (FDG) positron emission tomography/computed tomography (PET/CT) scan showed no abnormal FDG uptake in the spinal cord and lungs (**Figure 1C**), leading him to believe that he had fully recovered and that there was no need to continue atezolizumab treatment and regular follow-up.

**Figure 2:**
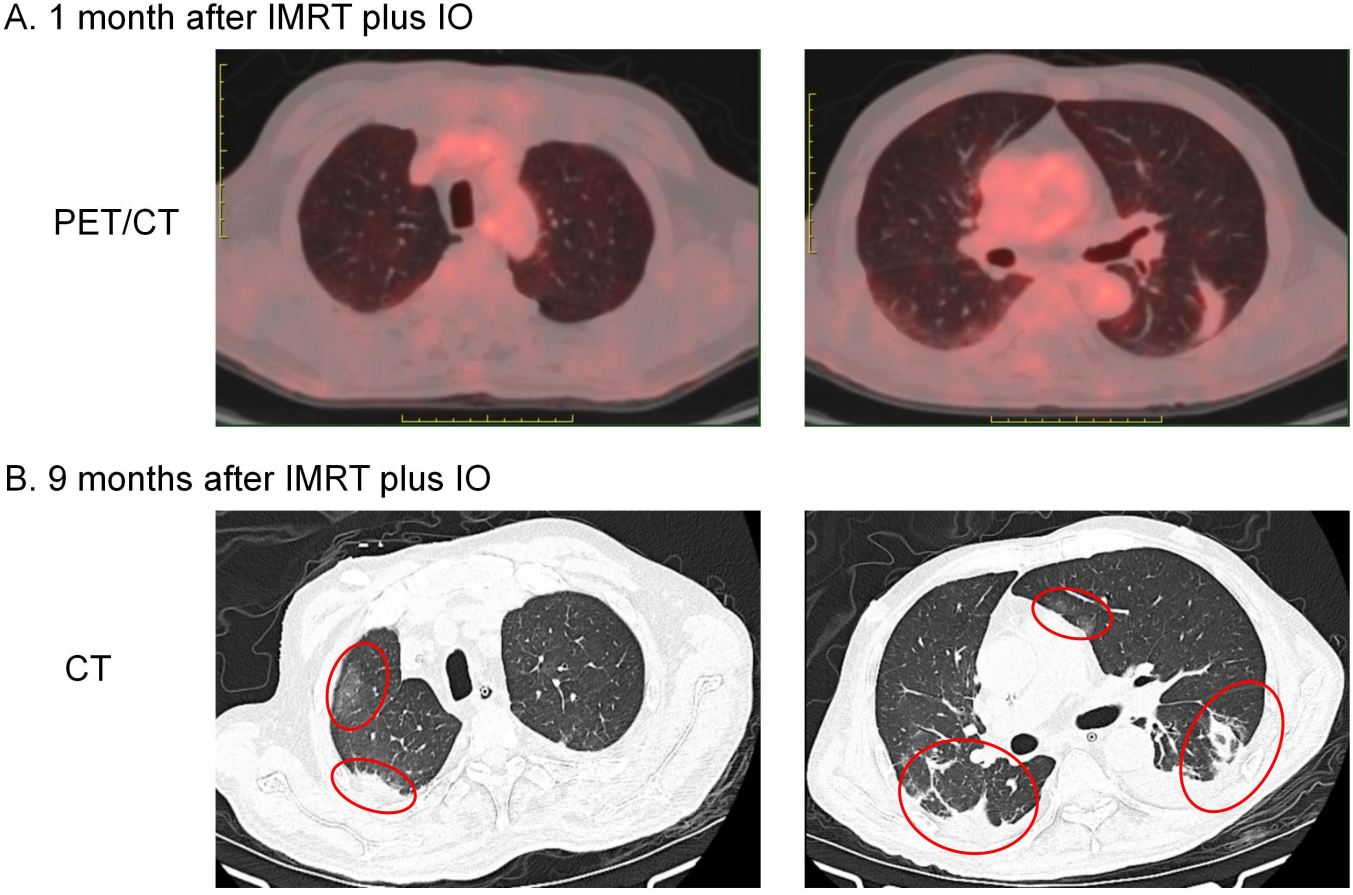
Imaging changes in bilateral lung lesions. **(A)** PET/CT performed 1 month after IMRT plus IO showed striated lesions without abnormal FDG uptake in the lower lobe of the left lung only. **(B)** Chest CT performed 9 months after IMRT plus IO showed bilateral pleural effusions with emerging lamellar exudative, semisolid multiple foci underpinned by old interstitial lung changes (indicated by red circles).

In February 2023 he was admitted to the respiratory unit of another hospital because of fever, cough and dyspnoea. Chest CT showed bilateral pleural effusions with emerging lamellar exudative, semisolid, multiple foci underpinned by old interstitial lung changes (**Figure 2**). Symptomatic treatment such as anti-infectives and respiratory support was given, but prolonged bed rest, malnutrition and cough weakness worsened his condition. Respecting his wishes, hospice care closes the book on him.

## 3 Discussion

About 40% of ISCMs are associated with BM (1), but spinal MRI is not routinely performed in patients with BM without spinal symptoms, so a proportion of ISCMs may be missed. This case suggests that spinal MRI should be better performed in patients with BM.

Optimal treatment modalities for ISCMs have not been established due to their rarity, heterogeneity, and rapid progression. Surgery can lead to a wide range of neurological complications (7, 22). In general, surgical resection has been proposed in the presence of good performance status (PS) or rapidly declining neurological function, or indolent and solitary spinal cord lesions without BM, or failure of radiotherapy, or the need to localise the primary malignancy (2, 23–25). However, patients with ISCMs often have poor PS and cannot tolerate surgery. Therefore, non-invasive therapies are more needed to control the progression of ISCMs. Radiotherapy alone can achieve an objective response rate (ORR) of 61.9% and a local control rate of 90.48%, which can maintain patients’ quality of life to some extent, although there is no evidence that radiotherapy can improve OS (1, 9). Chemotherapy is usually given in combination with radiotherapy, which is effective in a short term (11, 26). Data on biological therapies for ISCMs come mainly from three options that have been tested in the treatment of ISCMs-LC: targeted epidermal growth factor receptor (EGFR) mutants (13, 27), anaplastic lymphoma kinase (ALK) (28), and immune checkpoint inhibitors (ICIs) (29). However, these are all case reports and no large-scale clinical trials have been conducted.

**Figure 3:**
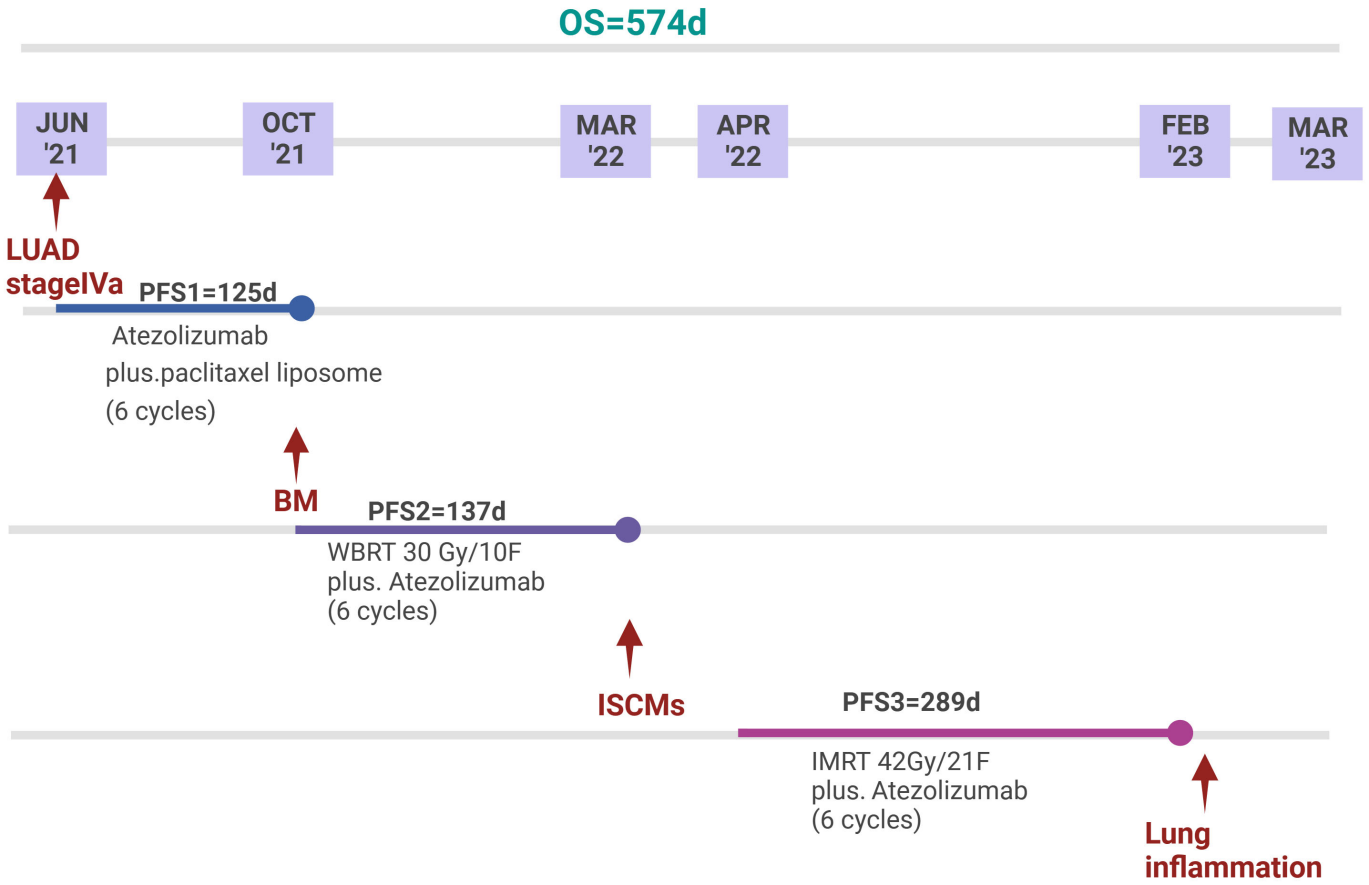
Timeline of anti-tumour treatment. Since the diagnosis of LUAD stage IVa, the patient achieved an overall survival of 574 days. Since the diagnosis of ISCMs, the patient achieved a PFS of 289 days. The patient eventually died of pneumonia.

For this patient, we maintained atezolizumab and added IMRT for the following reasons: 1. A case report demonstrated the potential efficacy of ICIs for small solitary parenchymal spinal cord metastases from NSCLC (29). 2. There is no evidence that changing other ICIs can improve ICI resistance. 3. Atezolizumab is active in a subset of patients with NSCLC with BM with acceptable toxicity (30, 31), and this patient had no significant adverse events during his atezolizumab therapy. 4. Although radiotherapy alone can achieve an ORR of 61.9% and a local control rate of 90.48%, there is no evidence that radiotherapy can improve OS (1, 9). A total dose of 42 Gy/21 fractions of IMRT combined with atezolizumab almost completely reversed the neurological deficits and achieved a relatively good survival of about 10 months, far exceeding the currently reported median survival (**Figure 3**). Therefore, this case illustrates the feasibility of IMRT combined with IO for ISCM.

The patient died of severe pneumonia, which taught us a painful lesson. He had received a total of 16 cycles of atezolizumab since 2021, and the last use of atezolizumab was approximately 8 months before the severe pneumonia. There was a lack of follow-up during the last 8 months, so it was difficult to track the time of onset of pulmonary symptoms and changes in lung imaging. In general, in patients who develop enlarged and increased lung lesions after IO, an exclusive diagnosis should first be made in combination with the medication history: 1. Factors of the tumor itself, such as tumor progression leading to intrapulmonary metastases or obstructive pneumonitis or carcinomatous lymphadenitis. 2. Factors of anti-tumor therapies, such as the interstitial changes of the lung induced by IO, targeted drugs, chemotherapy or radiotherapy. 3. Other non-tumor-related factors, such as pathogen infection, pulmonary embolism, cardiogenic pulmonary edema, progression of the underlying systemic disease, or progression of the underlying lung disease (e.g. worsening of interstitial fibrosis). This case highlights the importance of patient management during IO therapy and the importance of early recognition and management of immune-related adverse events.

## 4 Conclusion and future perspective

Due to new imaging techniques and the increasing life expectancy of patients with malignant tumors, more ISCMs will be encountered in the clinic (23). However, the lack of clinical trials for ISCMs makes optimized treatment strategies a puzzle. We reported that precision radiotherapy (such as IMRT) combined with IO showed potential efficacy against multifocal ISCMs, and future studies need to include more patients with multifocal ISCMs to explore the efficacy and safety of radiotherapy combined with IO.

## Data Availability

The original contributions presented in the study are included in the article/supplementary material. Further inquiries can be directed to the corresponding author.
